# Editorial: Nutrition and Regulation of Gastrointestinal Homeostasis, Injuries and Disturbances

**DOI:** 10.3389/fphys.2021.803200

**Published:** 2022-01-11

**Authors:** Daniele Maria-Ferreira, Elizabeth Soares Fernandes, Maria Fernanda de Paula Werner, Karien Sauruk da Silva, Bruna Carla da Silveira

**Affiliations:** ^1^Faculdades Pequeno Príncipe, Instituto de Pesquisa Pelé Pequeno Príncipe, Curitiba, Brazil; ^2^Programa de Pós-graduação em Biotecnologia Aplicada à Saúde da Criança e Do Adolescente, Faculdades Pequeno Príncipe, Curitiba, Brazil; ^3^Department of Pharmacology, Federal University of Parana, Curitiba, Brazil

**Keywords:** gastrointestinal homeostasis, gastrointestinal injuries, gastrointestinal disturbances, gastrointestinal disorders, dietary nutrients, natural polysaccharides

Gastrointestinal (GI) homeostasis is vital for maintaining animal and human health (MacDonald et al., 2011). In physiological conditions, complex regulatory pathways ensure the interaction of the host with external and endogenous factors that could cause some damage (Garrett et al., 2010). GI homeostasis is then established and maintained by a functional epithelial barrier, a tolerant immune response, and a commensal microbiota providing a healthy intestinal environment (Zheng et al., 2020). However, defects in any of these mechanisms, including exposure to exogenous invading pathogenic microorganisms (e.g., bacteria, fungi, and viruses), mechanical trauma, use of medications, among others, can favor an inadequate host response and the establishment of a harmful process, resulting in the development of GI injuries as well as systemic disorders (Toor et al., 2019).

Four articles were published in the Nutrition and Regulation of Gastrointestinal Homeostasis, Injuries and Disturbances Research Topic, which have been viewed more than 4,000 times. The articles published in this collection have provided new information on the most diverse and interesting subjects, from mechanical trauma on the gastrointestinal tract to nutrigenomics, effects of natural carbohydrates on the epithelial barrier function, to broiler intestinal health.

Two of the articles were mini-reviews. The one by Felisbino et al. discussed the effects of two types of diets: (i) the Mediterranean diet and (ii) a diet rich in polyphenols, on the expression of human genes related to type 2 diabetes mellitus (T2DM). The review by Sauruk da Silva et al. reviewed the recent advances on the potential of polysaccharides isolated from natural sources to protect the intestinal mucosa in non-clinical models of intestinal mucositis.

Felisbino et al. presented information on specific polyphenols with the ability to prevent or delay the onset of T2DM. Interestingly, the regular consumption of resveratrol, quercetin, genistein, catechins, or curcumin itself or, either a Mediterranean diet, can reduce the risk of T2DM by interacting with the genome. The main beneficial effects of polyphenol consumption included control of inflammation and oxidative stress, protection against cardiovascular diseases, reduction of glucose levels and dyslipidemia, in addition to protection of pancreatic β-cells. The interaction between polyphenols and the epigenome occurs mainly by enhancing demethylation and decreasing methylation of the DNA, by triggering acetylation of histone H3, and hypermethylation of genes. Despite the relative novelty and the scarce specific information on this subject in the literature, the authors managed to compile important data and relate them to T2DM.

Sauruk da Silva et al. summarized all articles available in the literature which used models of intestinal mucositis induced by antineoplastic therapy to investigate the beneficial effects of natural polysaccharides or polysaccharide extracts. The most used animal models to mimic intestinal mucositis were described in the context of treatment dosing schemes, duration, and routes of administration of chemotherapies. The authors also presented the different classifications of polysaccharides based on their structures and sources and made available in an attractive manner to the reader, the mechanisms of action of these macromolecules in the light of the intestinal mucositis. This mini-review provided important information and indicated that regardless of the polysaccharide source or type when given orally, these macromolecules can prevent intestinal mucositis through different mechanisms including the regulation of the inflammatory process and oxidative stress, and maintenance of the integrity of the intestinal epithelial barrier. In addition, the review also compiled relevant data from experimental models which can be further explored in the context of new therapeutic alternatives for this disease.

In addition to these two mini-reviews, two original articles were also published in this Research Topic. In a well-designed study by Liu et al., the non-directional mechanical trauma that often occurs in accidents - an important point within the context of injuries to the GI, was addressed. By using male-specific pathogen-free Sprague Dawley rats, the authors demonstrated that a non-directional rotational trauma model causes morphological changes along the GI tract (stomach, ileum, and cecum), at 12, 24, and 48. The cecal mucosa presented greater damage in comparison to those of the stomach and ileum. Twenty minutes after trauma, the cecal epithelial cell junction presented with significant enlargement, followed by an aggravation of the mucosal injury. Interestingly, LPS and D-lactic acid plasma concentrations were significantly higher at the same time point, when compared to the control group. The mechanisms by which mechanical trauma occurs in the gastrointestinal and, subsequently contribute to the development of secondary and more serious damage, are yet to be completely elucidated. Thus, this study author was of great contribution to the field. The indication that the cecal mucosa is a key location of bacterial translocation after mechanical trauma to the GI opens new venues for early interventions which may be able to prevent secondary injuries, including secondary myocardial infarct and even heart and multiple organ failure.

Finally, Xu et al., carried out a study with broilers and investigated whether supplementation with *Clostridium butyricum* (a probiotic) mitigates the effects of necrotic enteritis caused by *C. perfringens*. The authors evaluated animal growth, intestinal barrier function and morphology, and immunological parameters. Dietary supplementation with *C. butyricum* enhanced weight gain and decreased feed conversion rate in comparison with *C. perfringens*-infected animals. Also, *C. butyricum* supplementation improved broiler production performance; in addition to reducing the colonization of *C. perfringen*, as well as the intestinal damage caused by this pathogen. The study is very interesting. It not only brings novel information on the beneficial effects of *C. butyricum* in intestinal health but also indicates that this probiotic can potentially reduce *C. perfringens* infection and necrotic enteritis in chickens, thus, preventing economic losses in broiler poultry farming.

There is still significant scientific work to be done to refine our understanding of GI homeostasis, injuries, and disturbances to better guide the handling and management of humans and animals. Therefore, we would like to immensely thank all the authors who took the time to gather such interesting and varied information on the subject, as well as the reviewers who provided important contributions to the published manuscripts.

## Author Contributions

The editorial board members contributed equally to editing this collection. KS and BS equally contributed to the interpretation and summarization of the Research Topic information. All authors contributed to the article and approved the submitted version.

## Funding

This work was supported by the Conselho Nacional de Desenvolvimento Científico e Tecnológico (CNPq; 305676/2019-9 and 408053/2018-6), Instituto de Pesquisa Pelé Pequeno Príncipe, and INCT-INOVAMED.

## Conflict of Interest

The authors declare that the research was conducted in the absence of any commercial or financial relationships that could be construed as a potential conflict of interest.

## Publisher's Note

All claims expressed in this article are solely those of the authors and do not necessarily represent those of their affiliated organizations, or those of the publisher, the editors and the reviewers. Any product that may be evaluated in this article, or claim that may be made by its manufacturer, is not guaranteed or endorsed by the publisher.
